# Merkel Cell Carcinoma: An Update and Review

**DOI:** 10.3390/cancers15051534

**Published:** 2023-02-28

**Authors:** Eggert Stockfleth

**Affiliations:** Department of Dermatology, Venereology and Allergology, St. Josef Hospital, Ruhr University Bochum, Gudrunstrasse 56, 44791 Bochum, Germany; eggert.stockfleth@klinikum-bochum.de

Merkel cell carcinoma (MCC) is a rare, very aggressive skin cancer with a high mortality rate and a high tendency of metastatic spread [1]. MCC mainly affects elderly and immunosuppressed individuals. Thus, in a population with a growing life expectancy and consequent age-related immunological impairment, MCC cases are likely to increase in the near future. Merkel cell polyomavirus (MCPyV) infection, UV radiation and the impairment of cellular immunity have been identified as important factors in MCC development [2,3,4], but the oncogenic mechanisms in MCC are still not fully understood. However, the exploration of carcinogenic mechanisms is of pivotal importance with respect to the development of preventive and therapeutic measures.

As MCCs are frequently misdiagnosed, biomarkers are needed to improve their detection, in order to prompt biopsy to confirm diagnosis by histology and immunohistochemistry, respectively. In addition, biomarkers are required for prognostic evaluation, including for the patient-specific risk of metastatic spread, as well as for decisions about treatment. Recently, immune checkpoint inhibitors (ICI) have significantly improved the management of advanced MCC, with durable response rates in many (50–70%), but not all, metastatic cases [5]. Considering the high costs of ICI therapy and the potential development of various immune-related adverse events, there is an urgent need to characterize biomarkers for the indication and monitoring of specific therapies, finally resulting in a personalized management of MCC patients.

In this Special Issue of *Cancers*, entitled “Merkel Cell Carcinoma: An Update and Review”, the interested reader is provided with concise review articles reflecting the current knowledge of MCC epidemiology, pathogenesis and T cell immunity. In addition, clinical studies, including patient profiles with primary MCC and rare manifestations of MCC—such as nodal MCC and cardiac metastases—are included. The issue of MCC treatment is covered in several contributions and includes the evaluation of current treatment modalities, as well as the characterization of potential new drugs that may be used for MCC in the future. Finally, the topic of molecular markers (biomarkers) that may be used for prognostic evaluation or to guide MCC therapy is addressed in some articles.

The article series starts with a review by Silling et al. presenting an update on the epidemiology of MCPyV and MCC, as well as the characteristic features and risk factors of MCC. The authors report on the two entities of MCC, UV radiation-induced and MCPyV-induced, and summarize the differences and similarities in pathogenesis as well as prevalence variations in different geographical regions and patient populations. Studies on MCPyV detection and viral gene expression in skin malignancies other than MCC were also reviewed. Lower prevalence, lower viral load and the rarely detected expression of large T antigens do not support a causal role of MCPyV in non-MCC skin cancer [6].

The association of MCPyV with other malignancies was also addressed by Dimitraki and Sourvinos, who reviewed studies about the presence of the virus in various cancer types of different human organ systems [7]. As in non-MCC skin cancers, MCPyV DNA has been detected in the tissues of different tumours, but again with low prevalence and in low amounts. The authors conclude that the results of these studies do not provide evidence for a substantial role of the virus in the pathogenesis of malignancies other than MCC.

Based on studies in other cancers indicating the uptake of extracellular citrate by a plasma membrane variant of the mitochondrial citrate transporter pmCIC supporting cell proliferation, Drexler et al. examined the role of citrate homeostasis in MCC [8]. They report novel interesting data about citrate and pmCIC in the biology and pathogenesis of MCC. The proliferation of MCC tumour cells is stimulated by extracellular citrate, and this effect can be blocked by the inhibition of pmCIC using Na-gluconate. These findings characterize pmCIC as an attractive therapeutical target.

The important role of T cells in MCC pathogenesis was illustrated in a comprehensive review of Ouyang et al. [9]. In particular, the impact of tumour-infiltrating lymphocytes (TILs)—including different T cell subtypes—in tumour initiation, progression and clearance, was outlined, and interacting factors of the TME were discussed. Based on the present knowledge of the T cell immunity of MCC, the authors highlight the importance of understanding T cell-mediated reactions to design effective strategies of immunotherapy.

Following these articles on epidemiology and pathogenesis, the next papers of the Special Issue mainly consider the clinical aspects of MCC. Gualdi et al. report data from six Italian dermatological clinics regarding the primary diagnosis of MCC, including clinical and demographic data. The physicians primarily involved in the MCC assessment belong to various medical specialties and differ with respect to the initial management of patients, indicating a lack of standardization and the need for strategies to increase awareness among physicians, in order to improve MCC diagnosis [10].

Whereas MCC typically manifests as a skin nodule, rare cases may present with just a lymph node localization without a cutaneous primary site and no distant metastases (nodal MCC-UP). Fazio et al. present in their paper informative data from a single centre about the management of this rare MCC phenotype. Their findings suggest favourable outcomes (relapse-free survival and cancer-specific survival) of adjuvant radiotherapy after curative surgical treatment with lymphadenectomy [11].

Cardiac metastases of MCC represent another unusual manifestation, as distant metastases of MCC are mainly located in non-regional lymph-nodes, skin, liver and bone [12]. In the article of Akaike et al. [13], nine cases of cardiac metastatic MCCs were identified in a registry of almost 600 patients with metastatic MCC. Despite the small number of cases, the detailed evaluation with respect to clinical presentation, course of disease, therapy/management and prognosis suggests that the survival of patients with cardiac metastases is not worse than that of patients with metastases at other sites, and that patients with cardiac metastases show a good response to immunotherapy and cardiac radiotherapy.

The efficiency of postoperative radiotherapy and the role of regional lymph node irradiation in localized MCC is addressed in the study of Dinges et al. [14]. They report data from a single-centre retrospective analysis comparing patients with and without lymph node irradiation (LNI). Relapses mainly occurred in regional lymph nodes, and regional relapse was more frequent in patients without LNI, indicating that the inclusion of regional lymph nodes in postoperative radiotherapy may reduce the risk of regional relapse.

In another paper on MCC treatment, Dudzisz-Śledź et al. present the findings of a prospective multi-centre study from Poland [15]. Therapies currently used in four polish cancer centres to treat locally advanced MCC are unsatisfactory, and patients have a poor prognosis. The authors emphasize the need to improve the management of these patients in reference centres with experienced multidisciplinary teams available.

Although the management of advanced MCC has been improved by the introduction of immune checkpoint inhibitors, a significant portion of patients do not benefit from immunotherapy, indicating the need for further treatment options [16]. There are several new drugs in the pipeline for future MCC therapy. Among them, fumaric acid esters represent potentially useful compounds. Gambichler et al. studied the anti-neoplastic effects of dimethylfumarate (DMF) in MCPyV-negative MCC cell cultures and provide functional data concerning the mode of action as a precondition for potential clinical application in the future [17].

Finally, the Special Issue concludes with two papers dealing with the characterization of biomarkers that support disease management. By comparing the transcriptomes of primary MCC from patients with good and poor prognosis, Sundqvist et al. identified several differentially expressed genes, mainly involved in pathways promoting tumour progression (death-associated) and immune responses (survival-associated) [18]. These dysregulated genes may provide targets for future therapeutic interventions and potentially represent biomarkers for the prognostic evaluation and initiation of specific treatments.

The value of circulating tumour DNA (ctDNA) for the management of MCC patients was addressed in a review of Gao et al. [19]. Although the number of published studies on ctDNA in MCC is still small, ctDNA detection may have the power to become a useful tool to identify patients that may require specific therapies and to monitor the treatment response. Whereas commercial assays are already available for tumour agnostic testing, the use of tailored assays to detect patient-specific tumour mutations is still challenging and requires the identification of tumour-associated mutations by comprehensive investigations, such as whole-exome sequencing, which is not easily implemented in routine laboratories.

As with all rare diseases, rare tumours such as MCC also face the problem of insufficient awareness and are, consequently, afflicted by under- or misdiagnosis and inadequate treatment. In addition, it is less attractive for pharmaceutical industries to develop new therapeutical compounds for diseases of infrequent occurrence. With this Special Issue of *Cancers*, we hope to disseminate the present knowledge on MCC and improve the awareness of this rare skin cancer. We should, however, keep in mind that with the increasing life expectancy of the population, MCC is also likely to increase and may not be as rare in the future.
